# Wellens’ syndrome: incidence, characteristics, and long-term clinical outcomes

**DOI:** 10.1186/s12872-022-02560-6

**Published:** 2022-04-16

**Authors:** Li Zhou, Xuhe Gong, Tianhui Dong, He-he Cui, Hui Chen, Hongwei Li

**Affiliations:** 1 Department of Cardiology, Cardiovascular Center, Beijing Friendship Hospital, Capital Medical University, Yong’an Road, Xichen, Beijing, 100050 People’s Republic of China; 2 Department of Internal Medicine, Medical Health Center, Beijing Friendship Hospital, Capital Medical University, Beijing, 100050 People’s Republic of China; 3 Beijing Key Laboratory of Metabolic Disorder Related Cardiovascular Disease, Beijing, 100069 People’s Republic of China

**Keywords:** Wellens’ syndrome, NSTE-ACS, MACCE, Cardiac death, Angioplasty

## Abstract

**Background:**

Few studies with large sample sizes are available regarding patients with Wellens’ syndrome. Therefore, we sought to assess the current incidence, risk factors, clinical presentation and long-term outcomes of this population.

**Methods:**

Among a total of 3528 patients with ACS who underwent angioplasty from 2017 to 2019 in our centre, 2127 NSTE-ACS patients with culprit LAD vessels were enrolled in this study. According to electrocardiographic criteria, the patients were divided into a Wellens’ group (*n* = 200) and non-Wellens’ group (*n* = 1927). The primary endpoint was cardiac death; the secondary endpoint was MACCE, a composite of all-cause death, cardiac death, recurrent myocardial infarction, target lesion revascularization, heart failure and stroke.

**Results:**

The incidence of Wellens’ syndrome was 5.7% (200 of 3528) of all ACS patients. Wellens’ syndrome more often manifested as NSTEMI (69% *vs.* 17.5%, *P* < 0.001). The percentages of preexisting coronary heart disease (39.6% *vs.* 23%) and previous PCI (19.5% *vs.* 9%) were significantly higher in the non-Wellens’ group than in the Wellens’ group (all *P* < 0.001). More importantly, the proportion of early PCI was higher in the Wellens’ group (68% *vs.* 59.3%, *P* = 0.017). At a median follow-up of 24 months, Wellens’ syndrome was not associated with an increased risk of MACCE (*P* = 0.05) or cardiac death (*P* = 0.188).

**Conclusions:**

The presence of Wellens’ syndrome is not definitively associated with adverse prognosis in patients with NSTE-ACS. Age ≥ 65 years, diabetes, NSTEMI, eGFR < 60 ml/min and left main disease are associated with the incidence of cardiac death. Early recognition and aggressive intervention are critical, as they may help to attenuate adverse outcomes.

## Introduction

Acute coronary syndrome (ACS) remains a leading cause of mortality and morbidity worldwide, need for emergency care and eventual hospitalization [1–3]. The diagnosis of ACS relies on clinical history, electrocardiographic (ECG) changes, and cardiac biomarkers; but within the spectrum of ACS, subtle presentations exist that cannot be overlooked. Wellens’ syndrome is one such example, in which a patient can present with ECG changes that are not classic for myocardial ischaemia and even with negative cardiac biomarkers. As a well-known high-risk ACS, Wellens’ syndrome, first described by de Zwaan and Wellens in 1982 [4], is the characteristic ST-T segment change in the precordial leads, indicating a critical stenosis high in the left anterior descending arterial (LAD). Identifying the syndrome carries significant diagnostic and prognostic value [5]. According to the Fourth Universal Definition of myocardial infarction [6], absence of ST-elevation in the precordial leads, the symmetrical and often deep (> 2 mm) T wave inversions in the anterior precordial leads are an early sign that may precede the elevation of the ST-segment. Thus, this syndrome has been considered an acute ST-elevation myocardial infarction (STEMI) equivalent [7].

Patients with Wellens’ syndrome have an increased risk for extensive anterior wall myocardial infarction, and early coronary revascularization is essential in the management of these cases. These individuals constitute a special cohort with their own clinical characteristics, which may affect the outcomes in this population. However, the limited literature on the syndrome consists of mostly sporadic case reports and clinical experience. There is still a paucity of recent data on patients with Wellens’ syndrome. Updated information on the incidence, risk factors, angiographic findings and prognosis of this subset of patients should be taken into consideration when taking care of these patients.

The aims of this retrospective control study were therefore to investigate the incidence and the risk factor profile in Wellens’ syndrome patients versus other ACS patients with culprit LAD vessels admitted to Beijing Friendship Hospital in China between 2017 and 2019 and to study the clinical presentation, treatment and long-term outcomes in these patients.

### Study methods

#### Study design and participants

**Fig. 1:**
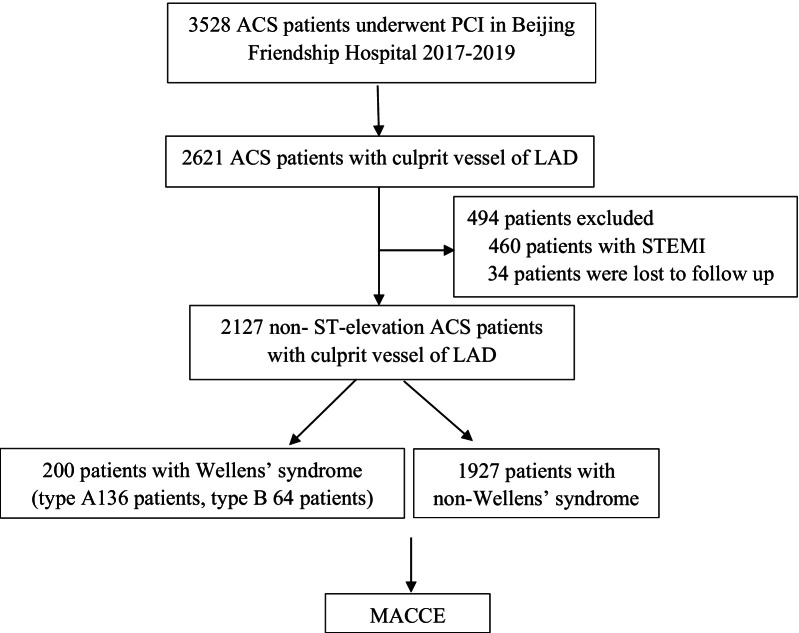
Flow chart of patient enrollment. *ACS*, acute coronary syndromes; *PCI*, percutaneous coronary intervention; *LAD*, left anterior descending arterial; *STEMI*, ST-elevation myocardial infarction; *MACCE*, major adverse cardiac and cerebrovascular events

This retrospective study was based on data from the Cardiovascular Center of Beijing Friendship Hospital Data Bank. The protocol was approved by the ethics committee of Beijing Friendship Hospital (2021-P2-096-01). From January 2017 to December 2019, coronary angioplasty was performed in 3528 consecutive ACS patients at our centre, and a total of 2621 patients with culprit LAD vessels were enrolled in this study. To rule out pathological Q waves in the ECG, 460 patients with STEMI were excluded. In addition, 34 patients were lost to follow-up. Finally, a total of 2127 patients were included in the final analysis. Among the 2127 cases, 200 met the ECG criteria of Wellens’ syndrome, including 64 cases of type A, 136 cases of type B, all of which were shown angiographically to have significant LAD stenosis. Baseline characteristics, percutaneous coronary intervention (PCI) procedures, management, and long-term outcomes were collected from medical records and the data bank and then analysed. A flowchart of the patient enrolment is shown in Fig. 1.

In all patients, 12-lead ECGs were routinely obtained once daily while they were in the hospital. Additional ECGs were checked during and after new attacks of chest pain. The criteria for Wellens’ syndrome are as follows [8, 9]: (a) prior history of chest pain, (b) minimal or no elevation of cardiac enzymes, (c) insignificant ST-segment elevation usually (< 1 mm), (d) no loss of precordial R waves, (e) absent precordial Q waves and (f) biphasic T waves in leads V_2_ and V_3_, or asymmetric, often deeply inverted T waves in leads V_2_ and V_3_. Wellens’ syndrome can be divided into two different types according to the precordial T wave pattern that is seen during the pain-free period. In type A, there are biphasic T waves typically observed in V_2_ and V_3_. In Type B, which is the most common form, there are deep, negative T waves in leads V_2_ and V_3_.

#### Data collection and clinical outcomes

The patients’ demographic information and cardiovascular risk factors, including hypertension, dyslipidaemia, diabetes mellitus, chronic kidney disease (CKD), peripheral arterial disease (PAD), heart failure (HF), smoking history, preexisting coronary heart disease (CHD) and previous PCI, were retrospectively collected from medical records. Body mass index (BMI) was calculated by dividing weight in kilograms by height in metres squared (kg/m^2^). Smoking history was defined as regularly smoking one or more cigarettes daily or smoking cessation within the past 12 months. Left ventricular ejection fraction (LVEF) and end diastolic dimension (EDD) were measured by transthoracic echocardiology before PCI. The angiogram data, including the number of stenosed coronary vessels, left main (LM) disease and PCI strategy, were obtained by reading the surgical report.

The primary endpoint for this analysis was cardiac death, defined as death caused by myocardial infarction, HF, or arrhythmia and unexplained sudden death [10]. The secondary endpoints were major adverse cardiovascular and cerebrovascular events (MACCEs), a composite of all-cause death, cardiac death, recurrent myocardial infarction, target lesion revascularization, HF, and stroke. Recurrent myocardial infarction was defined by the Fourth Universal Definition [6].

All MACCEs were thoroughly analysed and confirmed by two separate cardiologists simultaneously. Follow-up information after patient discharge from the hospital was obtained by clinic visits or phone interviews every 1–3 months, which were recorded in the data bank.

## Statistical analysis

Continuous variables are expressed as the mean ± standard deviation or as the median with interquartile range; one-way analysis of variance was used to compare differences between continuous variables. Categorical variables are expressed as percentages and were analysed using Pearson’s χ^2^ test or Fisher’s exact test of variance. The cumulative incidence was estimated by the Kaplan–Meier method, and differences between groups were assessed by the log-rank test [11].

Cox regression was used to estimate relative risks among groups of patients. All factors showing significance in the univariate analysis (*P* < 0.05) or an indicator clinically considered to be important for the outcome were then examined by a multivariate analysis. The results are reported as adjusted hazard ratios (*HRs*) with associated 95% confidence intervals (*CIs*). All statistical tests were two-tailed, with statistical significance defined as a *P* value of < 0.05. All analyses were performed by using SPSS (version 25.0, Chicago, IL, USA); Kaplan–Meier survival curves were generated with GraphPad Prism software (version 5; GraphPad, Inc., San Diego, CA) [11].

## Results

### Baseline characteristics

Patients stratified by Wellens’ syndrome characteristics are summarized in Table 1. The study cohort included 2127 NSTE-ACS patients with culprit LAD vessels. Of these patients, 200 (9.4%) had Wellens’ syndrome. Wellens’ syndrome most often manifested as non-ST-elevation myocardial infarction (NSTEMI) (69% *vs.* 17.5%, *P* < 0.001). The percentages of preexisting coronary heart disease (39.6% *vs.* 23%) and previous PCI (19.5% *vs.* 9%) were significantly higher in the non-Wellens’ group than in the Wellens’ group (all *P* < 0.001). The two groups of patients had a similar prevalence of sex distribution, hypertension, diabetes mellitus and hyperlipemia. There were also no significant differences in the medical history of prior myocardial infarction, HF, CKD, PAD or stroke. Compared with non-Wellens’ patients, more Wellens’ patients had a lower BMI (25.21 ± 2.97 *vs.* 25.93 ± 3.45 kg/m^2^, *P* = 0.005), waist circumference (90.79 ± 9.84 *vs.* 92.5 ± 10.08 cm, *P* = 0.025) and LVEF (0.62 ± 0.09 *vs.* 0.65 ± 0.08, *P* = 0.002). However, the LDL-C and EDD were higher in the Wellens’ group.

**Table 1:** Comparison of the baseline characteristics among patients

Variables	Wellens’ (N = 200)	Non-Wellens’ (N = 1927)	*P* value
Age (years)	63.3 ± 10.4	64.2 ± 9.7	0.158
Male	146 (73)	1314 (68.2)	0.163
Days	6 (5.8)	6 (4.7)	**< 0.001**
BMI (kg/m^2^)	25.21 ± 2.97	25.93 ± 3.45	**0.005**
WC (cm)	90.79 ± 9.84	92.5 ± 10.08	**0.025**
Smoking	114 (57)	1017 (55.8)	0.255
HT	133 (66.5)	1353 (70.2)	0.276
DM	70 (35)	810 (42)	0.055
Hyperlipemia	100 (50)	1030 (53.5)	0.352
History of CHD	46 (23)	763 (39.6)	**< 0.001**
Prior MI	15 (7.5)	199 (10.3)	0.206
HF	1 (0.5)	13 (0.7)	0.771
CKD	10 (5)	71 (3.7)	0.355
PAD	14 (7)	202 (10.5)	0.121
Stroke	39 (19.5)	317 (16.5)	0.271
Previous PCI	18 (9)	376 (19.5)	**< 0.001**
NSTEMI	138 (69)	338 (17.5)	**< 0.001**
EDD (cm)	5.19 ± 0.56	5.08 ± 0.52	**0.006**
LVEF	0.62 ± 0.09	0.65 ± 0.08	**0.002**
LDL-C (mmol/L)	2.5 ± 0.73	2.25 ± 0.75	**< 0.001**
eGFR (ml/min)	86.69 ± 24.76	88.09 ± 22.2	0.408
HBA1c (%)	6.54 ± 1.46	6.7 ± 21.45	0.159
Multivessel disease	177 (88.5)	1712 (88.8)	0.884
LM disease	18 (9)	229 (11.9)	0.226
Plain balloon angioplasty	7 (3.5)	279 (14.6)	**< 0.001**
Drug-eluting stent	193 (96.5)	1635 (84.8)	**< 0.001**
Stent length < 30 mm	58 (29)	542 (28.1)	0.794
Number of stents	1.35 ± 0.63	1.14 ± 0.69	**< 0.001**
Early PCI (< 48 h after first presentation)	136 (68)	1143 (59.3)	**0.017**

Bold values indicate that the difference is statistically significant (*P* < 0.05)

Data are presented as absolute numbers and percentages (for categorical variables) or mean value ± SD (for continuous variables) unless otherwise specified

*BMI*, body mass index; *WC*, waist circumference; *HT*, hypertension; *DM*, diabetes mellitus; *CHD*, coronary heart disease; *MI*, myocardial infarction; *HF*, heart failure; *CKD*, chronic kidney disease; *PAD*, peripheral arterial disease; *PCI*, percutaneous coronary intervention; *NSTEMI*, non-ST‑elevation myocardial infarction; *EDD*, left ventricular end diastolic dimension; *LVEF*, left ventricular ejection fraction; *LDL-C*, low-density lipoprotein cholesterol; *eGFR*, estimated glomerular filtration rate (calculated via Modification of Diet in Renal Disease equation); *HbA1c*, glycated hemoglobin, *LM*, left main

Table 1 also shows the differences in angiographic characteristics and treatment data between Wellens’ and non-Wellens’ patients. The two groups had a similar rate of LM disease and multivessel disease. Compared to the non-Wellens’ group, Wellens’ patients had more drug-eluting stents implanted (96.5% *vs.* 84.8%, *P* < 0.001) and greater stent numbers (1.35 ± 0.63 *vs.* 1.14 ± 0.69, *P* < 0.001) but less plain balloon angioplasty (3.5% *vs.* 14.6%, P < 0.001). More importantly, the proportion of early PCI (< 48 h after first presentation) was higher in Wellens’ group (68% *vs.* 59.3%, *P* = 0.017).

### Clinical outcomes

**Fig. 2:**
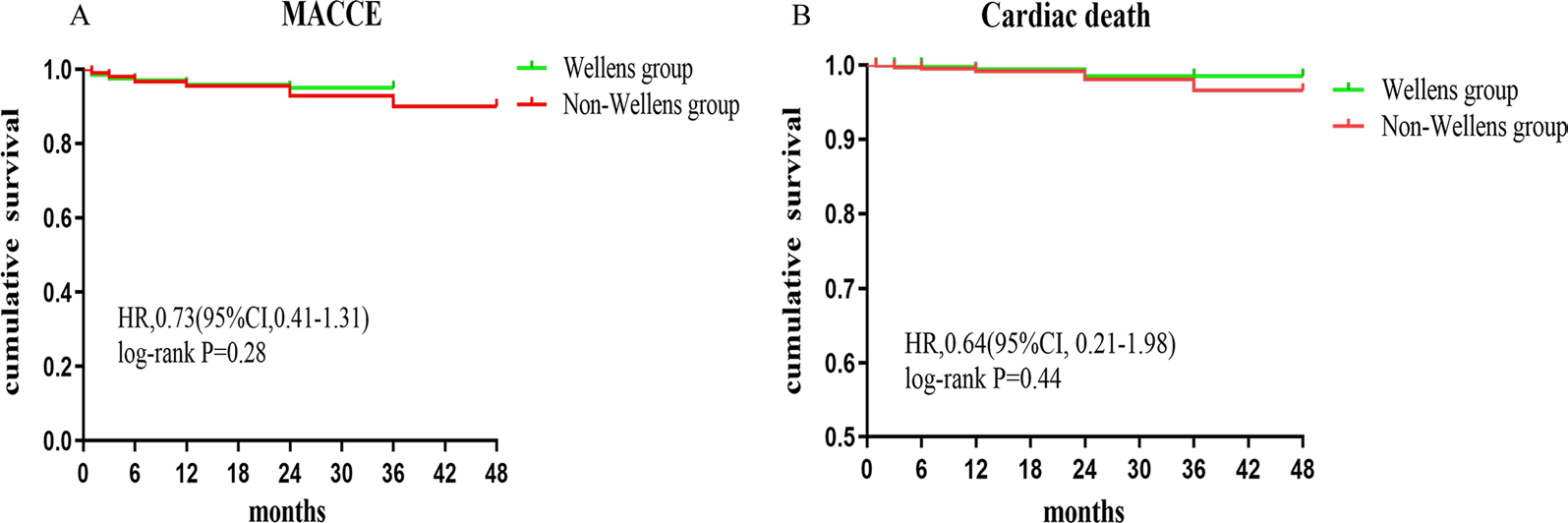
Kaplan–Meier analysis of MACCE (secondary endpoint, panel **A**) and cardiac death (panel **B**) for overall patients stratified by Wellens’ syndrome (green line) and non-Wellens’ syndrome (red line)

The median follow-up time was 24 months. The clinical outcomes in the Wellens’ and non-Wellens’ groups are shown in Table 2. Compared with Wellens’ group, the non-Wellens’ group had significantly higher rates of rehospitalization (19.3% *vs.* 10.5%, *P* = 0.002). There were no differences in MACCE (Wellens’ 4.5% *vs.* non-Wellens’ 6.5%, *P* = 0.26), all-cause death, cardiac death, HF, target vessel revascularization, recurrent myocardial infarction or stroke. Kaplan–Meier analysis also revealed no difference in MACCE (*P*_log-rank_ = 0.28) or cardiac death (*P*_log-rank_ = 0.44) between the Wellens’ and non-Wellens’ groups (Fig. 2). Univariate and multivariate Cox regression analysis results of the effect of Wellens' syndrome on clinical outcomes in NSTE-ACS patients are shown in Table 3. Wellens’ syndrome did not directly affect MACCEs, cardiac death or all-cause death.

**Table 2:** Comparison of clinical outcome between Wellens’ and non-Wellens’ group

Variables, n (%)	Wellens’(N = 200)	No-Wellens’ (N = 1927)	*P* value
MACCE	9 (4.5)	126 (6.5)	0.26
All-cause death	4 (2)	58 (3)	0.419
Cardiac death	2 (1)	34 (1.8)	0.425
Re-hospitalization	21 (10.5)	372 (19.3)	0.002
Heart failure	1 (0.5)	25 (1.3)	0.329
Target vessel revascularization	0	18 (0.9)	0.17
Recurrent myocardial infarction	5 (2.5)	48 (2.5)	0.994
Stroke	2 (1)	21 (1.1)	0.907

Values are n (%)

*MACCE*: major adverse cardiac and cerebrovascular events, a composite of all-cause death, cardiac death, recurrent myocardial infarction, target vessel revascularization, heart failure, and stroke

**Table 3:** Univariate and multivariate Cox regression analysis results of Wellens' on Clinical outcome in NSTE-ACS patients

	Univariate		Multivariate	
	HR (95%CI)	*P* value	HR (95%CI)	*P* value
MACCE	0.7 (0.35–1.37)	0.293	0.5 (0.25–1)	0.05
All-cause death	0.68 (0.25–1.87)	0.451	0.48 (0.17–1.37)	0.171
Cardiac death	0.57 (0.14–2.39)	0.444	0.38 (0.09–1.61)	0.188

Compared with the non-Wellens’ group, Hazard ratios for events in Wellens’ group

### Predictors of survival

**Fig. 3:**
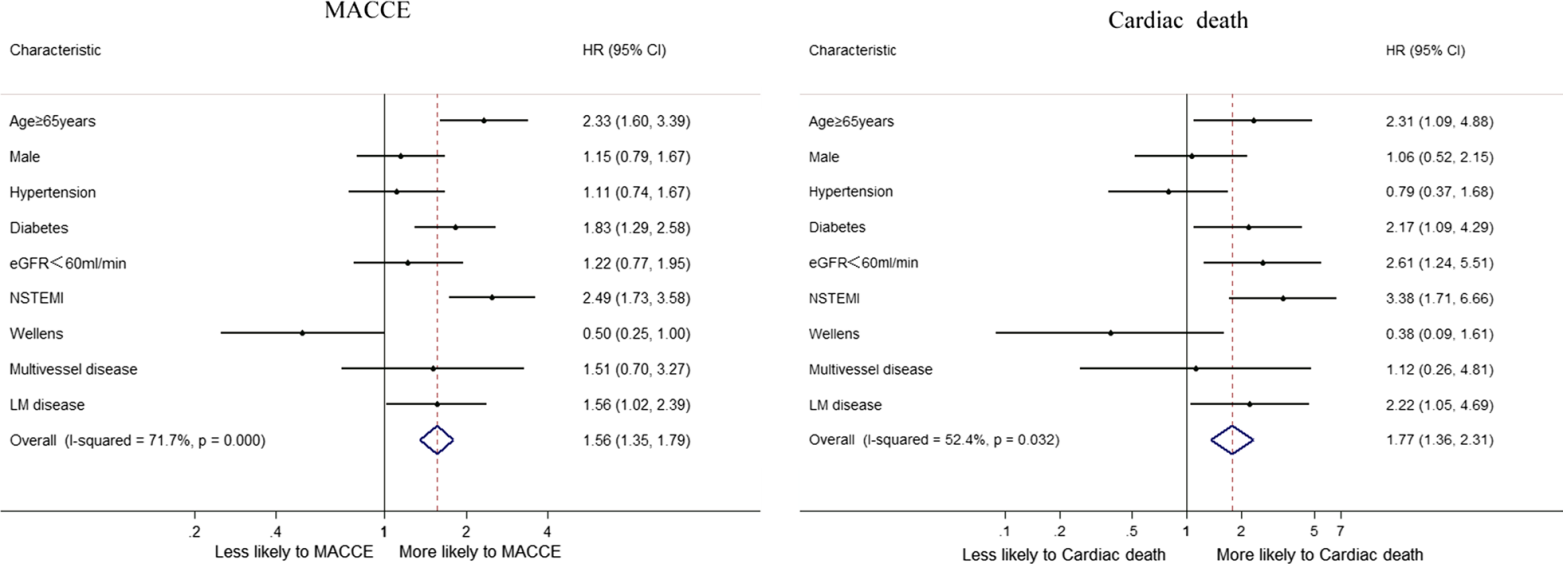
Factors independently associated with MACCE and cardiac death in overall patients in multivariable Cox regression analysis

Considering all patients, a multivariable Cox regression analysis (Table 4) was used to identify clinical and angiography independent predictors of cardiac death and MACCE. For MACCEs, the final multivariable mode included age ≥ 65 years, diabetes mellitus, NSTEMI and LM disease. For cardiac death, the final multivariable mode included age ≥ 65 years, diabetes mellitus, NSTEMI, eGFR < 60 ml/min and LM disease. Overall, Wellens’ syndrome was not associated with an increased risk of MACCEs (*P* = 0.05) or cardiac death (*P* = 0.188). In Fig. 3, NSTEMI was the biggest influencing factor for poor MACCEs (*HR:* 2.49, 95% *CI:* 1.73–3.58) and cardiac death (*HR:* 3.38, 95% *CI:* 1.71–6.66).

**Table 4:** Multivariate Cox regression analysis in the overall patients

Predictor variable	HR (95% CI)	*P* value
MACCE		
Age ≥ 65 years	2.33 (1.6–3.39)	< 0.001
Male	1.15 (0.79–1.67)	0.468
HT	1.11 (0.74–1.66)	0.606
DM	1.83 (1.29–2.58)	0.001
eGFR < 60 ml/min	1.22 (0.77–1.95)	0.396
NSTEMI	2.49 (1.73–3.58)	< 0.001
Wellens’ syndrome	0.5 (0.25–1)	0.05
Multivessel disease	1.5 (0.69–3.26)	0.302
LM disease	1.56 (1.02–2.39)	0.04
Cardiac death		
Age ≥ 65 years	2.31 (1.09–4.88)	0.029
Male	1.06 (0.52–2.15)	0.883
HT	0.79 (0.37–1.68)	0.534
DM	2.16 (1.09–4.29)	0.027
eGFR < 60 ml/min	2.61 (1.24–5.51)	0.012
NSTEMI	3.38 (1.71–6.66)	< 0.001
Wellens’ syndrome	0.38 (0.09–1.61)	0.187
Multivessel disease	1.11 (0.26–4.8)	0.884
LM disease	2.22 (1.05–4.69)	0.037

*HR*, Hazard ratio; *CI*, Confidence interval; *MACCE*, major adverse cardiac and cerebrovascular events; *HT*, hypertension; *DM*, diabetes mellitus; *NSTEMI*, non-ST‑segment elevation myocardial infarction; *LM*, Left main

## Discussion

Wellens’ syndrome is a pattern of precordial T-wave abnormalities, first described by Wellens and his group, representing critical LAD coronary artery stenosis. In Wellens’ initial study group of 145 patients admitted for unstable angina, 9% had the typical pattern upon presentation, with a further 9% developing T-wave changes within 24 h. Seventy-five percent of these patients went on to suffer an anterior wall myocardial infarction despite the relief of symptoms with medical therapy. In a second prospective study, 14% of patients with unstable angina met the ECG criteria, all of whom were shown angiographically to have significant LAD stenosis [12].

In our study, patients with Wellens’ syndrome represented 5.7% (200 of 3528) of ACS patients hospitalized for three years. More specifically, the incidence of Wellens’ syndrome in NSTE-ACS patients with culprit LAD vessels was 9.4% (200 of 2127). Of these patients, 64 cases presented with Type A Wellens’ syndrome, which comprises 32% (64 of 200) of cases and shows biphasic T waves in leads V_2_ and V_3_. The remaining 68% (136 of 200) had Type B Wellens’ syndrome, which shows deeply inverted, symmetrical T waves in predominantly V_2_ and V_3_. This percentage is roughly in line with previous findings, which showed that type A Wellens’ constitutes roughly 24% of cases and that type B Wellens’ accounts for the remaining 76% of cases [9]. Thus, we confirmed that type B Wellens’ syndrome is more common.

The mechanism of Wellens’ syndrome remains unclear. It is considered a preinfarction stage of CHD, as the T-wave changes in the syndrome usually occur during the pain-free period [13, 14]. It is also postulated that the changes in the ECG account for reperfusion of the ischaemic myocardium due to alleviation of spasm of the proximal LAD artery [15–17]. There is also a view that the syndrome may be related to myocardial stunning or myocardial hibernation.

Multiple risk factors are common in patients with Wellens’ syndrome, and the majority of these patients are reported to have at least one traditional cardiovascular risk factor. In our study, patients with Wellens’ syndrome did not differ from the non-Wellens’ group in terms of factors such as hypertension, diabetes mellitus, and dyslipidaemia. More than half of the patients with Wellens’ syndrome were current smokers (57%). Compared with non-Wellens’ patients, they had higher LDL-C levels (2.5 ± 0.73 *vs.* 2.25 ± 0.75 mmol/L, *P* < 0.001) but were less likely to be diagnosed with hyperlipemia (50% *vs.* 53.5%, *P* = 0.352). Patients with Wellens’ syndrome were also less likely than non-Wellens’ patients to have a history of CHD and previous PCI at admission, which means that Wellens’ syndrome tends to occur in patients with new-onset cardiovascular disease. In addition, there were no significant differences between the two groups regarding comorbidities, such as HF, CKD, PAD, and stroke.

NSTEMI was the clinical presentation in 69% of Wellens’ patients, which was much higher than the 17.5% in the non-Wellens’ group. However, previous studies have suggested that unstable angina is the main clinical manifestation in Wellens’ patients [4, 12]. The increased incidence rate of NSTEMI should be due to the adoption of the Fourth Universal Definition of Myocardial Infarction defined by elevated cardiac troponin, especially hs-cTn, which indicates myonecrosis.

The angiographic characteristics of CHDin patients with Wellens’ syndrome may differ from the presentation in other ACS patients. Although there were no differences in LM disease and multivessel disease between the two groups, Wellens’ patients had more drug-eluting stents implanted and greater stent numbers than non-Wellens’ patients. This discrepancy may, to a large degree, be explained by a higher percentage of proximal and middle LAD lesions in patients with Wellens’ syndrome. Balloon angioplasty, including plain old balloon angioplasty and drug-coated balloon angioplasty, is not appropriate for these critical sites [18–20].

It is well known that patients with Wellens’ syndrome are at high risk of extensive anterior wall infarction, which might lead to serious left ventricular dysfunction, malignant arrhythmias, and sudden death. To our surprise, there was no statistically significant difference in MACCEs between the two groups during the follow-up period (mean 24 months), even though the Wellens’ group had a higher rate of myocardial infarction at admission. Furthermore, the 2-year incidence of cardiac death was similar in the two groups (Wellens’: 1% *vs.* non-Wellens’: 1.8%, *P* = 0.425). We believe that this low incidence was obtained by our approach, which was to treat these patients aggressively with urgent angiography and intervention. Data showed that the proportion of early PCI was higher in the Wellens’ group (68% *vs.* 59.3%, *P* = 0.017). This highlights the importance of timely identification of Wellens’ syndrome and appropriate management in this group of patients. Most patients, when identified early and taken for cardiac catheterization, do well after appropriate intervention. Regarding the strength of the improvement in medical procedures, a small number of Wellens’ patients (10.5%) were readmitted after PCI for 2 years, which was much lower than that of the non-Wellens’ group (19.3%, *P* = 0.002). We did not find a direct correlation between Wellens’ syndrome and adverse prognosis in patients with NSTE-ACS. NSTEMI was the biggest influencing factor for poor MACCEs and cardiac death. This suggests that the prognosis of CHD depends on its severity.

Early PCI is currently the preferred treatment for patients with high-risk NSTE-ACS [21, 22]. However, few patients in the real world can receive such treatment within the 24 h recommended by the guidelines, especially those patients who are pain-free at admission. In our study, we presumed that an aggressive invasive strategy (< 48 h after first presentation) could attenuate the risk of MACCE and avoid long-term adverse outcomes in patients with Wellens’ syndrome.

The main strengths of this study are the large and unselected population comprising nearly all NSTE-ACS patients with culprit LAD vessels treated at our hospital from 2017 to 2019 and the nearly complete follow-up. However, this was a single-centre, retrospective observational study. Therefore, the choice of therapeutic strategy reflected the convention and tendency of our single centre, which may affect the objectivity of the conclusions. Further prospective multicentre studies are needed to validate our findings.

## Limitations

The following limitations were present in this study. (1) Lack of more information on angiographic and procedural characteristics of the study population. (2) Although we used multivariate Cox regression analysis to adjust for differences in baseline characteristics, there may still be unknown confounding factors. Therefore, the research results should be cautiously interpreted. (3) As the follow-up time was short, the long-term effect of Wellens’ syndrome has yet to be determined.

## Conclusions

First, our study revealed that the incidence of Wellens’ syndrome can reach up to 5.7% as assessed by coronary arteriography in clinical practice. Second, Wellens’ patients have a lower prevalence of a history of CHD and previous PCI at admission; thus, Wellens’ syndrome is more common in populations with new-onset cardiovascular disease. Third, the presence of Wellens’ syndrome is not definitively associated with adverse prognosis in patients with NSTE-ACS. Early recognition and aggressive intervention are critical, as they may help to attenuate adverse outcomes.

## Data Availability

The datasets generated and/or analysed during the current study are not publicly available due to database principle but are available from the corresponding author on reasonable request.

## Abbreviations

ACS: Acute coronary syndromes
MACCE: Major adverse cardiac and cerebrovascular events
NSTEMI: Non-ST-elevation myocardial infarction
PCI: Percutaneous coronary intervention
CHD: Coronary heart disease
LM: Left main disease
LAD: Left anterior descending arterial
LCX: Left circumflex artery
RCA: Right coronary artery
CKD: Chronic kidney disease
PAD: Peripheral arterial disease
HF: Heart failure
EDD: Left ventricular end diastolic dimension
LVEF: Left ventricular ejection fraction
LDL-C: Low-density lipoprotein cholesterol
eGFR: Estimated glomerular filtration rate
BMI: Body mass index
HR: Hazard ratio
CI: Conference interval
